# Development and external validation of the SMA2SH2ERS risk prediction model for aneurysmal subarachnoid haemorrhage in the general population: a population-based prospective cohort study

**DOI:** 10.1136/bmjopen-2024-091756

**Published:** 2025-01-15

**Authors:** Vita M. Klieverik, Jos P. Kanning, Ina L. Rissanen, Kristiina Rannikmae, Amy E. Martinsen, Bendik S. Winsvold, Mirjam I. Geerlings, Ynte M. Ruigrok

**Affiliations:** 1 Neurology and Neurosurgery, University Medical Centre Utrecht, Utrecht, Netherlands; 2 Department of Neurology and Neurosurgery, University Medical Center Utrecht, Utrecht, Netherlands; 3 University Medical Centre Utrecht, Utrecht, Utrecht, Netherlands; 4 Usher Institute, University of Edinburgh, Edinburgh, UK; 5 University of Oslo, Oslo, Norway; 6 Department of Public Health and Nursing, Norwegian University of Science and Technology, Trondheim, Norway; 7 FORMI, Oslo Universitetssykehus, Oslo, Norway; 8 Department of General Practice, Amsterdam UMC Locatie AMC, Amsterdam, Noord-Holland, Netherlands

**Keywords:** Cardiovascular Disease, Intracerebral Haemorrhage, Stroke

## Abstract

**ABSTRACT:**

**Objectives:**

Aneurysmal subarachnoid haemorrhage (ASAH) is a severe stroke type, preventable by screening for intracranial aneurysms followed by treatment in high-risk individuals. We aimed to develop and validate a risk prediction model for ASAH in the general population to identify high-risk individuals.

**Design:**

We used the population-based prospective cohort studies of the United Kingdom (UK) Biobank for model development and the Trøndelag Health (HUNT) Study for model validation.

**Participants:**

Participants missing data were excluded. A total of 456 856 individuals from the UK Biobank and 46 483 individuals from the HUNT Study were included.

**Primary and secondary outcome measures:**

Incident ASAH identified using the International Classification of Diseases codes, ICD-9 430 and ICD-10 I600 to I609 codes.

**Results:**

In the development cohort, ASAH occurred in 738 (0.2%) during 5 407 909 person-years of follow-up. We developed a multivariable Cox regression model to identify predictors for ASAH. Predictive performance was assessed using discrimination and calibration, and we corrected for overfitting using bootstrapping techniques. Predictors for ASAH were sex (S), diabetes mellitus (M), age and alcohol consumption (A^2^), smoking (S), hypertension and hypercholesterolaemia (H^2^), educational attainment (E), regular physical activity (R) and family history of stroke (S; SMA^2^SH^2^ERS), and multiple interactions between these predictors. The concordance statistic (c-statistic) of the model in the development cohort was 0.62 (95% CI 0.60 to 0.64). Predicted absolute 10-year ASAH risk varied from 0.042% to 0.52%. In the validation cohort, 220 individuals developed ASAH, and the c-statistic of this model was 0.64 (95% CI 0.58 to 0.69). Both models showed reasonable calibration.

**Conclusions:**

Our SMA^2^SH^2^ERS model provides ASAH risk estimates between 0.042% and 0.52% for the general population. While overall ASAH risk is low, the model identifies individuals with up to 12 times increased risk compared with those at the lowest risk.

**STRENGTHS AND LIMITATIONS OF THIS STUDY**

- Large prospective cohort with extensive sample size and follow-up data derived from the International Classification of Diseases codes, enabling robust model development.
- External validation in an independent population cohort, enhancing model generalizability and practical utility.
- Predictors readily accessible to general practitioners during routine consultations, facilitating easy integration into practice.
- The United Kingdom (UK) Biobank participants are more likely to be women, older individuals and of higher socioeconomic status compared with non-participants.
- Uncertainty on the accuracy of incident aneurysmal subarachnoid haemorrhage identification in population cohorts like the UK Biobank.

## Introduction

 Unruptured intracranial aneurysms (UIAs) affect 3% of the general population.^1^ When a UIA ruptures, it causes an aneurysmal subarachnoid haemorrhage (ASAH), striking at a mean age of 55 years and more often in women than men.^2^ The incidence of ASAH is 6.1 per 100 000 person-years, corresponding to a lifetime risk of 0.2%.^3^ Approximately one-third of patients with ASAH die, and half of the survivors require continuous care,^4^ often with severe cognitive impairments affecting functionality and quality of life.^2^ ASAH presents a significant socioeconomic burden comparable with ischaemic stroke in potential life years lost.^5^

Approximately 24% of patients with ASAH die before receiving medical attention, with early effects of ASAH being the leading cause of death among those admitted to the hospital.^2 6^ Consequently, opportunities to improve prognosis after ASAH are limited, making prevention essential to reducing the disease burden. Non-invasive screening for UIAs followed by endovascular or neurosurgical treatment can prevent ASAH.^7^ Screening is already proven cost-effective for first-degree relatives of patients with ASAH.^8 9^ Individuals with one affected first-degree relative with ASAH have an estimated lifetime risk of ASAH of up to 0.4%,^10^ and in 4% of these individuals, UIAs can be identified at first screening.^11^ In individuals with two or more affected first-degree relatives, the estimated lifetime risk increases to up to 10%^10^ with UIAs identified in 11% at the first screening.^12^ Whether additional high-risk individuals in the general population, regardless of a known family history of UIAs, may benefit from preventive screening is unclear. 

Risk factors for ASAH include female sex, older age, hypertension, smoking and alcohol consumption,^13^,^16^ but a comprehensive risk prediction model applicable to the general population is lacking. Estimating individual absolute ASAH risk in primary care settings could help identify high-risk candidates for UIA screening. Thus, we aimed to develop and externally validate a risk prediction model for ASAH in the general population to estimate individual absolute ASAH risk.

## Methods

The design and results of this study are reported following the Strengthening the Reporting of Observational Studies in Epidemiology guidelines and the Transparent Reporting of a Multivariable Prediction Model for Individual Prognosis or Diagnosis statement.

### Development cohort

For model development, we utilised data from the United Kingdom (UK) Biobank Prospective Cohort Study, a large population-based study with over 500 000 participants aged 37–73, recruited between 2006 and 2010.^17^ Data from the UK Biobank were linked to the International Classification of Diseases (ICD) codes, ICD-9 and ICD-10, to record diagnosis information, alongside national death and cancer registries. Follow-up data were available until 28 March 2021. All participants provided written informed consent, and the study was approved by the North West Multicentre Research Ethics Committee and the National Health Service National Research Ethics Service (ref 11/NEW/0382). Official approval for the present study was considered unnecessary.

### Validation cohort

For model validation, we utilised data from the Trøndelag Health (HUNT) Study, a general population-based cohort comprising over 240 000 Norwegian participants aged 20 or older, recruited between 1984 and 2008.^18^ HUNT Study data were linked to Hospital Episode Statistics and national health registries. We focused on data from the HUNT2 (recruitment 1995–1997) and HUNT3 (recruitment 2006–2008) studies, previously employed in a study on ASAH and UIA genetic risk.^19^ Participants with prior ASAH were excluded. Follow-up data were available until 6 June 2018. All participants provided written consent, and the study was approved by the Regional Committee for Medical Research Ethics (ref #2015/578).

### Outcome and candidate predictors

The primary outcome, incident ASAH, was identified using ICD-9 430 and ICD-10 I600 to I609 codes in both the UK Biobank and the HUNT Study. We included ICD-10 I608 and I609 codes, expected to encompass solely non-aneurysmal cases, comprising approximately 10–15% of all subarachnoid haemorrhage cases.^2^ However, these codes likely also include ASAH cases in the UK Biobank, as they represent 56.6% of the total cases (418 out of 738). Candidate predictors, chosen based on literature review, were limited to factors accessible to general practitioners, including sex, age, family history of stroke, hypertension, smoking status, hypercholesterolaemia, regular physical activity, hormone replacement therapy (HRT), diabetes mellitus (DM), alcohol consumption and educational attainment.^13^,^16^ A family history of stroke was defined as at least one first-degree relative being affected by the disease. Hypertension was defined as a systolic blood pressure of ≥140 mm Hg or a diastolic blood pressure of ≥90 mm Hg and/or use of antihypertensive medication. We categorised smoking status into (1) never smokers, (2) former smokers and (3) current smokers. Hypercholesterolaemia is defined as the use of cholesterol-lowering medication. Regular physical activity is defined as vigorous physical activity ≥three times per week. We grouped HRT into (1) never users and (2) former and current users combined. DM was defined based on a past medical history of DM and/or use of antidiabetic medication. We categorised alcohol consumption into (1) no alcohol consumption, (2) alcohol consumption on special occasions and (3) daily or almost daily alcohol consumption. We grouped educational attainment into (1) high, (2) intermediate and (3) low educational attainment. High educational attainment was defined as having a university or college degree, intermediate educational attainment as having either an advanced level qualification, ordinary level qualification, certificate of secondary education, national vocational qualification, higher national diploma, higher national qualification or other professional qualification, and low educational attainment as having no degree. While most predictors increase risk, some, like hypercholesterolaemia and DM, may decrease it.^13^,^16^ Family history of ASAH was omitted due to unavailability in the UK Biobank. Interaction terms (age with HRT, smoking with alcohol consumption, regular physical activity with hypertension, hypercholesterolaemia and DM) were included, alongside sex interaction terms with other predictors, to explore potential effect modification by sex.^20^ Definitions for these predictors are detailed in the Supplementary Material.

### Statistical analysis

Statistical analysis involved expressing normally distributed continuous variables as means±SD and skewed distributed continuous variables as medians with corresponding IQR. Distributional assumptions were verified visually using normal probability plots. Categorical variables were presented as counts with percentages. In the development cohort, missing data were minimal (ranging from 0.001% to 5.5%), and participants with missing data were excluded.^21^ No missing data were observed in the validation cohort.

For model development, multivariable Cox proportional hazards regression analysis was conducted using follow-up time as the time scale. Follow-up data were censored at the time of incident ASAH, date of death or last follow-up assessment on 28 March 2021, whichever came first. The functional form of continuous candidate predictor age was assessed using martingale residuals.^22^ Candidate predictors were considered for model entry regardless of their univariable association with incident ASAH. Backward selection based on the Akaike Information Criterion was performed to evaluate predictor contributions. Proportional hazard assumption was assessed visually and numerically using scaled Schoenfeld residuals plots and tests. To address overfitting, a shrinkage factor was applied to regression coefficients determined by bootstrapping procedures.^23^ Hazard ratios with corresponding 95% CI represented the estimated effect sizes of independent predictors.

Model performance was evaluated using discrimination and calibration. Discrimination, measured by the concordance statistic (c-statistic), was corrected for overoptimism through bootstrapping.^23^ Calibration indicates the agreement between predicted and observed probabilities of incident ASAH, visually using 5- and 10-year calibration plots.^24^ Statistical analyses were conducted using R statistical software, version 4.0.2, with an online interactive risk calculator developed using the Shiny R package (1.7.1). The risk calculator predicts an individual’s absolute ASAH risk at 5- and 10-year follow-up based on provided predictors, with each predictor’s contribution calculated by dividing regression coefficients by the smallest coefficient and rounding to the nearest integer.

### Patient and public involvement statement

Patients and/or the public were not involved in the design, conduct, reporting or dissemination plans of this research.

## Results

### Baseline characteristics

From the UK Biobank, 493 650 participants were recruited, with 456 856 included after excluding those with missing data (n=36 794). Among UK Biobank participants, 54.0% were women, the mean age was 56.4±8.1 years, and 738 (0.2%) developed ASAH during 5 407 909 person-years of follow-up (13.6 per 100 000 person-years, median follow-up 12.1 years, range from 2 days to 14.3 years; table 1). In the HUNT Study, 46 483 participants were included, with 53.1% women, mean age 59.1±14.1 years, and 220 (0.5%) developed ASAH during an estimated follow-up time of 1 993 060 person-years (11.0 per 100 000 person-years) based on data from a previous study that used the same HUNT Study.^25^

**Table 1:** Baseline characteristics of the development and validation cohorts

	Development cohort(n=4 56 856)		Validation cohort(n=46 483)	
**Characteristic**	n	%	n	%
Women	246 771	54.0	24 661	53.1
Age (years)				
<50	109 062	23.9	12 996	28.0
≥50	347 794	76.1	33 534	72.0
Mean±SD	56.4±8.1		59.1±14.1	
Family history of stroke	119 690	26.2	10 224	22.0
Hypertension	227 823	49.9	17 332	37.3
Smoking status				
Never smoking	250 870	54.9	18 808	40.5
Former smoking	159 174	34.8	16 207	34.9
Current smoking	46 812	10.2	11 468	24.7
Hypercholesterolaemia	77 510	17.0	13 152	28.3
Regular physical activity	148 892	32.6	2075	4.5
HRT	93 223	20.4	3393	7.3
Diabetes mellitus	22 903	5.0	1357	2.9
Alcohol consumption				
Never	34 201	7.5	670	1.4
On special occasions	327 445	71.7	37 813	81.3
Daily or almost daily	95 210	20.8	8000	17.2
Educational attainment				
Low	71 427	15.6	16 790	36.1
Intermediate	230 438	50.4	20 595	44.3
High	154 991	33.9	9098	19.6

HRT, hormone replacement therapy; NA, not available

### Model development and performance

**Figure 1:**
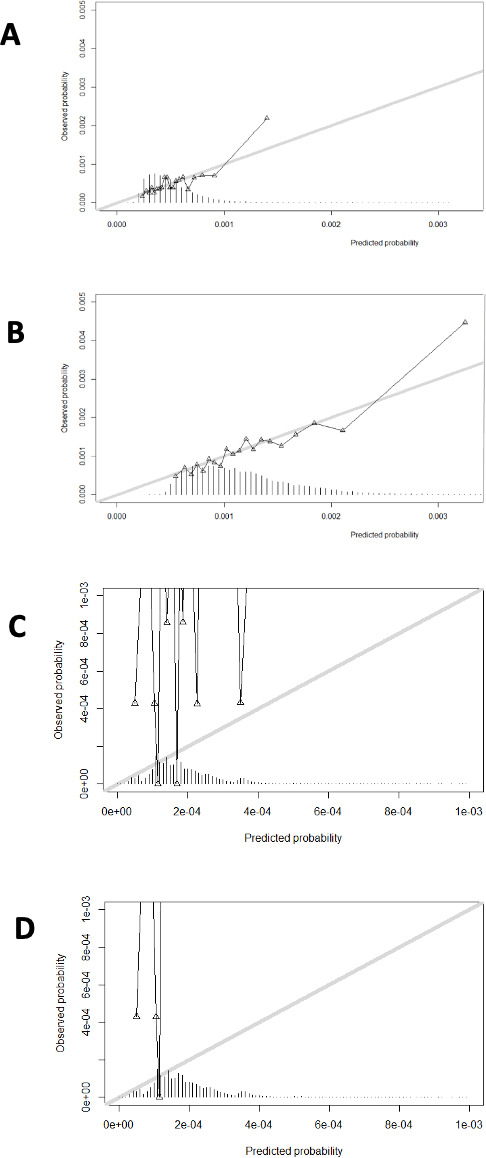
Calibration plots of predicted and observed probabilities for the development cohort at (**A**) 5 years and (B) 10 years and for the validation cohort at (C) 5 years and (D) 10 years.

Predictors were sex (S), DM (M), age and alcohol consumption (A^2^), smoking (S), hypertension and hypercholesterolaemia (H^2^), educational attainment (E), regular physical activity (R) and family history of stroke (S; SMA^2^SH^2^ERS), with multiple predictor interactions, including smoking-alcohol and sex with age, hypertension and smoking (table 2). HRT and the interactions between age and HRT, regular physical activity and hypertension, and sex with other predictors than age, hypertension and smoking were excluded due to limited predictive value. Age was analysed linearly, and proportional hazard assumptions were met (online supplemental figure 1). We inspected the scaled Schoenfeld residuals plots and tests for each independent predictor and detected no deviations from the proportional hazard assumption (online supplemental figure 1 and online supplemental table 1). Following shrinkage of the regression coefficients, the c-statistic of the model in the development cohort was 0.62 (95% CI 0.60 to 0.64). The c-statistic of the model in the validation cohort was 0.64 (95% CI 0.58 to 0.69). The 5- and 10-year calibration plots for the development and validation cohorts showed fair to good correspondence between predicted and observed risk (figure 1).

**Table 2:** Univariable and multivariable Cox proportional hazards regression analysis of predictors of incident aneurysmal subarachnoid haemorrhage

	Univariable	Multivariable*
**Predictor**	**HR (95% CI**)	**HR (95% CI**)
Female sex	1.50 (1.29 to 1.75)	0.40 (0.14 to 1.12)
Age per year	1.03 (1.02 to 1.04)	1.01 (1.00 to 1.03)
Family history of stroke	1.26 (1.08 to 1.47)	1.14 (0.99 to 1.31)
Hypertension	1.33 (1.15 to 1.54)	1.45 (1.15 to 1.84)
Smoking status		
Never smoking	Reference	
Former smoking	1.07 (0.91 to 1.26)	1.13 (0.87 to 1.47)
Current smoking	2.14 (1.76 to 2.60)	1.70 (1.22 to 2.37)
Hypercholesterolaemia	1.05 (0.86 to 1.27)	0.98 (0.79 to 1.21)
Regular physical activity	0.90 (0.77 to 1.06)	1.02 (0.88 to 1.18)
Diabetes mellitus	0.88 (0.61 to 1.26)	0.70 (0.47 to 1.06)
Alcohol consumption		
Never	1.56 (1.23 to 1.98)	1.41 (1.07 to 1.85)
On special occasions	Reference	
Daily or almost daily	1.14 (0.95 to 1.36)	1.13 (0.88 to 1.45)
Educational attainment		
Low	1.29 (1.07 to 1.56)	1.05 (0.88 to 1.24)
Intermediate	Reference	
High	0.75 (0.63 to 0.89)	0.84 (0.72 to 0.98)
Interactions		
Former smoking * never alcohol consumption	1.32 (0.78 to 2.24)	1.32 (0.84 to 2.10)
Former smoking * daily alcohol consumption	0.93 (0.62 to 1.40)	0.92 (0.64 to 1.31)
Current smoking * never alcohol consumption	0.44 (0.19 to 1.00)	0.47 (0.23 to 0.98)
Current smoking * daily alcohol consumption	0.97 (0.61 to 1.54)	1.02 (0.68 to 1.54)
Regular physical activity * hypercholesterolaemia	0.75 (0.48 to 1.19)	0.68 (0.44 to 1.04)
Regular physical activity * DM	1.68 (0.79 to 3.58)	1.93 (0.96 to 3.89)
Female sex * age per year	1.02 (1.00 to 1.04)	1.03 (1.01 to 1.04)
Female sex * hypertension	0.82 (0.60 to 1.13)	0.75 (0.56 to 1.00)
Female sex * former smoking	0.82 (0.58 to 1.15)	0.86 (0.63 to 1.16)
Female sex * current smoking	1.45 (0.96 to 2.20)	1.54 (1.06 to 2.22)

* The initial regression coefficients were corrected for overfitting with a shrinkage factor of 0.88.

.DMdiabetes mellitusHR, hazard ratios

### Individual risk prediction

To determine an individual’s absolute risk of ASAH, one can utilise the original regression equation from online supplemental table 2. However, due to the complexity of multiple predictor interactions, the manual calculation of absolute risk is challenging. Therefore, we developed an online interactive SMA^2^SH^2^ERS risk calculator, accessible at https://asah-prediction.shinyapps.io/app-1/, enabling the calculation of individual ASAH risks at 5- and 10 year follow-ups based on provided predictors. The mean predicted 5-year absolute ASAH risk was 0.05% (95% CI 0.0498% to 0.0503%), ranging from 0.018% (95% CI 0.016% to 0.021%) to 0.22% (95% CI 0.15% to 0.29%). Predicted 10-year cumulative absolute risk averaged 0.13% (95% CI 0.129% to 0.131%), ranging from 0.042% (95% CI 0.041% to 0.044%) to 0.52% (95% CI 0.35% to 0.68%). In the UK Biobank data, only 0.006% of participants (28 out of 456 856) had a predicted 10-year cumulative absolute risk exceeding 0.40% (the risk of ASAH in individuals with one first-degree relative with ASAH).

Among UK Biobank participants, the lowest absolute risk was for a 38-year-old non-smoking man with DM and hypercholesterolaemia, who consumes alcohol occasionally, exercises regularly and has high educational attainment. Conversely, the highest absolute risk was for a 73-year-old woman with hypertension and a family history of stroke, who is a former smoker, abstains from alcohol, exercises regularly and has low educational attainment.

## Discussion

We developed the SMA^2^SH^2^ERS risk prediction model to estimate individuals’ absolute ASAH risk using readily available predictors in primary healthcare. Independent predictors included sex (S), DM (M), age and alcohol consumption (A^2^), smoking (S), hypertension and hypercholesterolaemia (H^2^), educational attainment (E), regular physical activity (R) and family history of stroke (S; SMA^2^SH^2^ERS), with multiple predictor interactions, including three with sex (age, hypertension and smoking). This risk prediction model was developed for the general population, regardless of a known presence or absence of UIAs. Predicted 5-year absolute ASAH risk ranged from 0.018% to 0.22% and 10-year cumulative absolute risk ranged from 0.042% to 0.52%. While overall ASAH risk is low, the SMA^2^SH^2^ERS model identifies individuals with up to 12 times increased risk compared with those at the lowest risk.

To our knowledge, this is the first study to develop and validate a risk prediction model for ASAH in the general population. Two previous studies examined ASAH risk in patients with proven UIAs. In these studies, as in ours, age, sex and hypertension were identified as predictors.^14 24^

The finding that female sex is a predictor for ASAH aligns with prior studies indicating a higher risk in women, both in general populations and among patients with UIAs.^3 26^ UIAs are more prevalent in women, especially after age 50, coinciding with increased ASAH incidence in this demographic.^1 2^ This suggests an interaction between age and sex, potentially linked to hormonal changes during and postmenopause, theorised to elevate UIA risk.^27^ However, the precise role of female hormones in ASAH pathogenesis remains uncertain.^27 28^ Our examination of HRT revealed no independent association with ASAH risk. Literature on HRT’s role in ASAH is conflicting, with studies suggesting risk reduction, increased risk or neutral effects.^28 29^ These inconsistencies highlight the need for further investigation into HRT’s impact and other female hormonal factors on ASAH risk. Differential effects of risk factors based on sex may also contribute to this disparity. Previous research has shown that women with hypertension or who smoke are at greater ASAH risk than men with similar risk factors, a phenomenon corroborated by our study.^19 27^

We observed an increased risk of ASAH associated with familial stroke, a novel finding not previously demonstrated. We used this predictor as a proxy for familial ASAH, given its absence in our data. This aligns with prior research indicating familial ASAH as a risk factor, given ASAH’s classification as a stroke subtype.^7 10^ The increased ASAH risk in familial stroke is likely due to a combination of genetic and clinical risk factors, including hypertension and smoking.^29 30^ We also found an elevated risk of ASAH associated with alcohol abstinence, potentially linked to conditions that prevent alcohol consumption but also elevate ASAH risk.

### Strengths and limitations

A significant strength of our study lies in the large prospective cohort with follow-up data derived from ICD codes, enabling robust model development. The extensive sample size facilitated the examination of numerous candidate predictors and potential interactions. We also conducted external validation in an independent population cohort, enhancing the model’s generalisability and practical utility. Notably, the predictors in our model are readily accessible to general practitioners during routine consultations, facilitating easy integration into daily practice. Consequently, we opted against incorporating genetic risk factors into our model, as previous research has shown their limited added value over clinical data for ASAH prediction.^19^

Limitations include missing data in the development cohort, necessitating the exclusion of affected participants. However, the small proportion of missing data was anticipated to have a minimal impact. Another limitation stems from the use of the UK Biobank as our development cohort, which may skew towards more women, older individuals and higher socioeconomic status compared with non-participants.^31 32^ Nonetheless, we mitigated this by incorporating educational attainment as a proxy for socioeconomic status. Another limitation of the UK Biobank is that it lacked data for familial ASAH, so we had to use familial stroke as a proxy. This may have reduced the predictive performance of the model, as family history of ASAH is a known risk factor for ASAH.^10 12^ Moreover, the UK Biobank’s limited ethnic diversity precluded subgroup analyses to assess model validity across ethnicities, which may have reduced the generalisability of our model.^3 13 14^ However, validation in the HUNT study, which includes more participants with low education, partially addressed this limitation.^18^ The final limitation of using the UK Biobank was that the incidence of ASAH was higher in the development cohort than reported in the general population (13.6 per 100 000 person-years compared with 6.1 per 100 000 person-years, respectively), which may have resulted in a slight overestimation of the predicted risks. A limitation of using the HUNT study was that the incidence of ASAH per 100 000 person-years was unavailable in the validation cohort. Previous research has reported an ASAH incidence of 11.0 per 100 000 person-years in the validation cohort, the HUNT study.^25^ Additionally, the accuracy of incident ASAH identification in population cohorts like the UK Biobank remains uncertain, with limited data available. While a study assessing 24 ASAH cases reported a positive predictive value for ASAH ICD codes in the UK Biobank being 71% (95% CI, 49% to 87%),^33^ further research with larger ASAH patient cohorts is warranted to confirm these findings. Furthermore, uncertainty persists regarding whether the ICD codes used encompass solely ASAH or also include non-aneurysmal cases.^34^ We opted to include the ICD-10 I608 and I609 codes, indicative of non-aneurysmal cases, expecting them to account for 10–15% of all subarachnoid haemorrhage cases.^2^ However, in our study, their prevalence constituted a significantly larger proportion (418/738, 56.6%), suggesting they likely encompass ASAH cases as well. This supports our decision to include these codes. Sensitivity analysis excluding these ICD-10 I608 and I609 codes yielded a slightly higher c-statistic (0.72 (95% CI, 0.69 to 0.75) vs 0.62 (95% CI, 0.60 to 0.64)), although with reduced statistical power due to fewer cases. Lastly, the relatively low ASAH incidence contrasts with the prevalent predictors for this disease,^13^,^16^ which may limit the attainment of a very high c-statistic.

### Implications and future perspectives

Our SMA^2^SH^2^ERS risk prediction model offers insights into ASAH predictors in the general population, identifying individuals with up to a 12-fold increased risk compared with those at the lowest risk.

While the calibration plot demonstrated good correspondence between predicted and observed ASAH risk in the development cohort, there was less good calibration in the validation cohort. It seems that the predictor set in our current risk prediction model is inadequate for accurately predicting ASAH risk in the validation cohort. This discrepancy may arise from differences in ASAH epidemiology and candidate predictors in the validation cohort. Therefore, the SMA^2^SH^2^ERS risk prediction model in its current form is not suitable for use in clinical practice. Further validation in larger population-based cohorts and, if necessary, adjustment of the predictor set in the risk prediction model is required. Moreover, prior research indicates the cost-effectiveness of screening for UIAs in individuals with two or more first-degree relatives with ASAH, who have an estimated ASAH lifetime risk of up to 10%. Screening for UIAs is also likely to be cost-effective for those with one first-degree relative with ASAH, who have an estimated ASAH lifetime risk of 0.4%. Given our model’s ability to predict 10-year cumulative absolute risks up to 0.52%, it may aid in identifying high-risk individuals for preventive UIA screening. However, future studies must evaluate the cost-effectiveness as well as the net benefit of such screening for individuals identified as high risk by the SMA^2^SH^2^ERS model. Additionally, considering interactions between sex and other predictors, future investigations should explore separate risk prediction models for men and women. Lastly, further research on the differential effects of risk factors and female-specific ASAH risk factors is warranted.

## supplementary material

10.1136/bmjopen-2024-091756 online supplemental file 1

## Data Availability

Data are available upon reasonable request.
