# Supplementary material for: Development and external validation of the SMA2SH2ERS risk prediction model for aneurysmal subarachnoid haemorrhage in the general population: a population-based prospective cohort study
Source: BMJ Open. 2025 Jan 15;15(1):e091756. doi: 10.1136/bmjopen-2024-091756 (PMC11751821; doi:10.1136/bmjopen-2024-091756)
Supplement: online supplemental file 1 [file bmjopen-15-1-s001.docx]

**SUPPLEMENTAL MATERIALS**

**Development and external validation of the SMA^2^SH^2^ERS risk prediction model for aneurysmal subarachnoid haemorrhage in the general population**

Vita M. Klieverik^1,2^, MD, MSc; Jos P. Kanning^1,2^; MSc, Ina L. Rissanen^2^, MD, PhD; Kristiina Rannikmäe^3^, PhD; Amy E. Martinsen^4,5,6^, PhD; Bendik S. Winsvold^5,6,7^, MD, PhD; Mirjam I. Geerlings^2,8,9,10^, PhD; Ynte M. Ruigrok^1^, MD, PhD

**Affiliations**

1. Department of Neurology and Neurosurgery, University Medical Centre Utrecht Brain Centre, University Medical Center Utrecht, Utrecht, The Netherlands
2. Julius Center for Health Sciences and Primary Care, University Medical Center Utrecht and Utrecht University, Utrecht, The Netherlands
3. Centre for Medical Informatics, Usher Institute, University of Edinburgh, Edinburgh, UK
4. Institute of Clinical Medicine, University of Oslo, Oslo, Norway
5. K.G. Jebsen Centre for Genetic Epidemiology, Department of Public Health and Nursing, Faculty of Medicine and Health Sciences, Norwegian University of Science and Technology, Trondheim, Norway
6. Department of Research, Innovation and Education, Division of Clinical Neuroscience, Oslo University Hospital, Oslo, Norway
7. Department of Neurology, Oslo University Hospital, Oslo, Norway
8. Amsterdam UMC, location University of Amsterdam, Department of General Practice, Meibergdreef 9, Amsterdam, the Netherlands
9. Amsterdam Public Health; & Later life, and Personalized Medicine, Amsterdam, the Netherlands
10. Amsterdam Neuroscience; Neurodegeneration, and Mood, Anxiety, Psychosis, Stress, and Sleep, Amsterdam, the Netherlands

**Supplemental Figure 1** **Martingale r
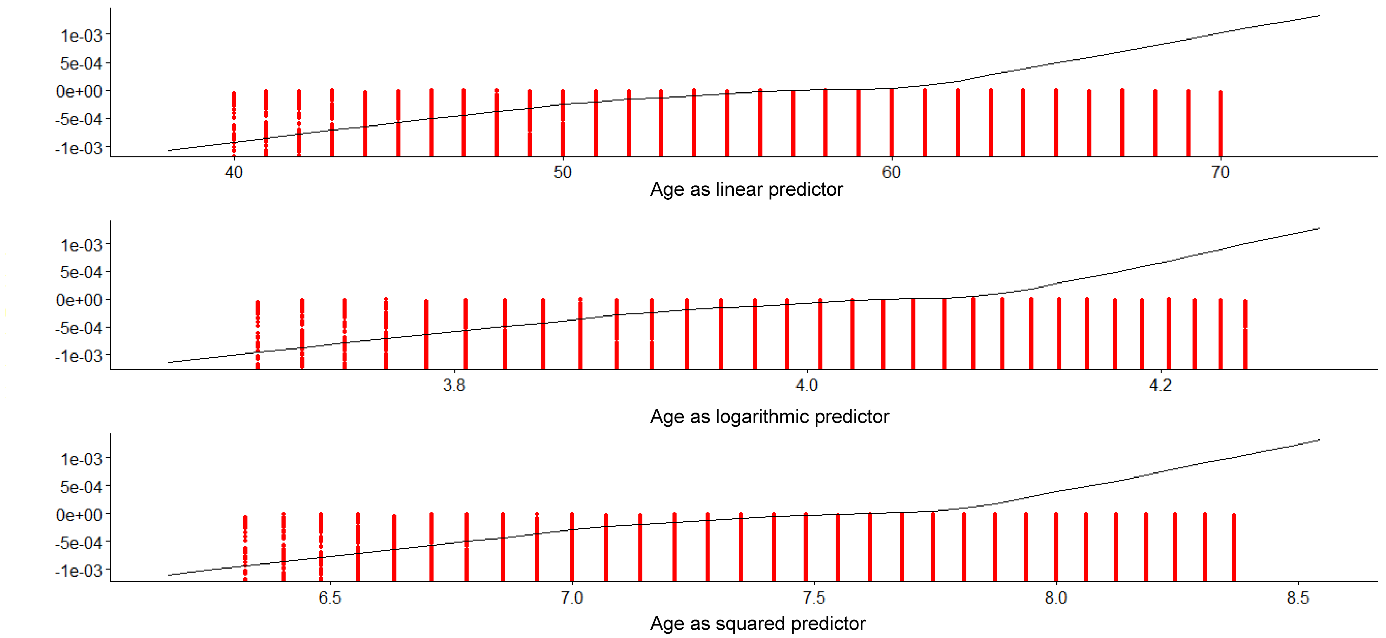
esiduals for the continuous predictor age.**

**Supplemental Figure 2A Scaled Schoenfeld residuals plot with survival time on linear time scale.**


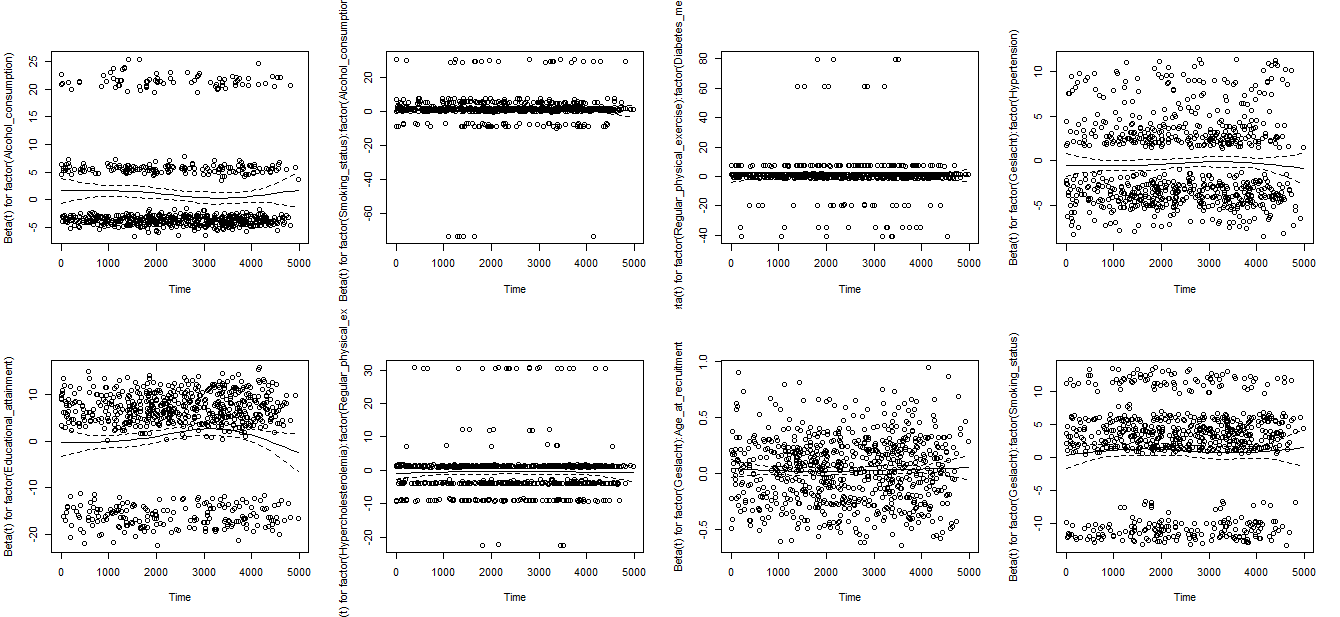


**Supplemental Figure 2B Scaled Schoenfeld residuals plot with survival time on Kaplan-Meier-transformed time scale.**


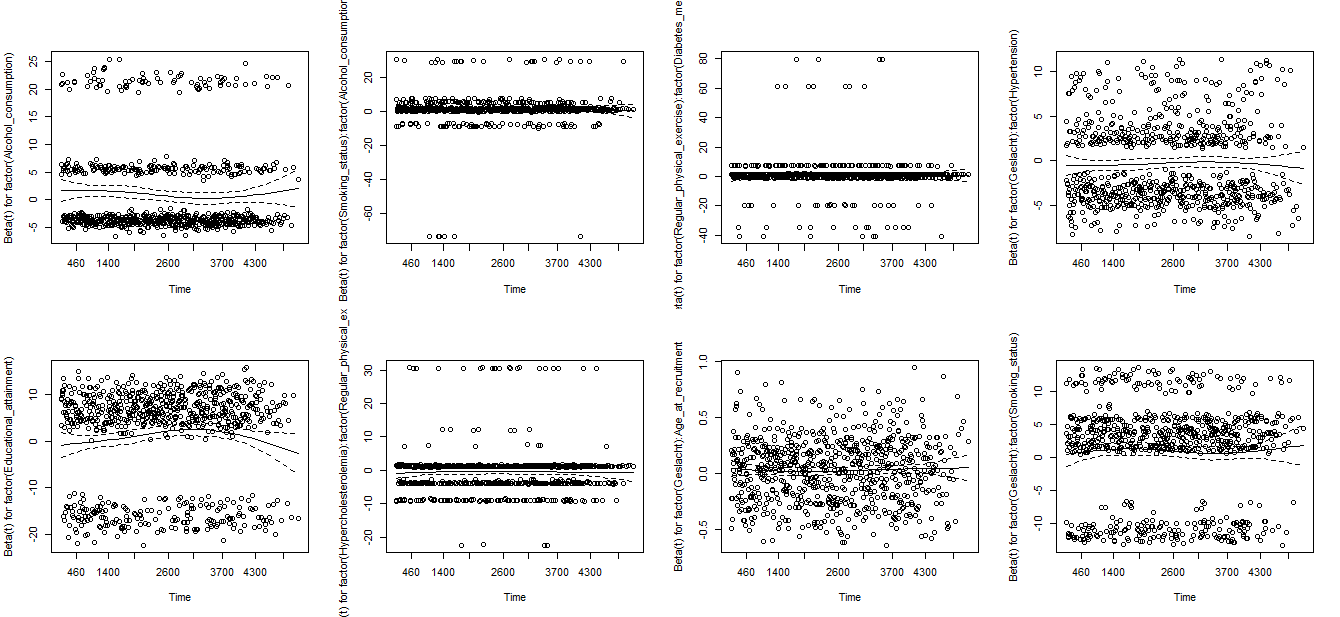


**Supplemental Figure 2C Scaled Schoenfeld residuals plot with survival time on logarithmic-transformed time scale.**

**
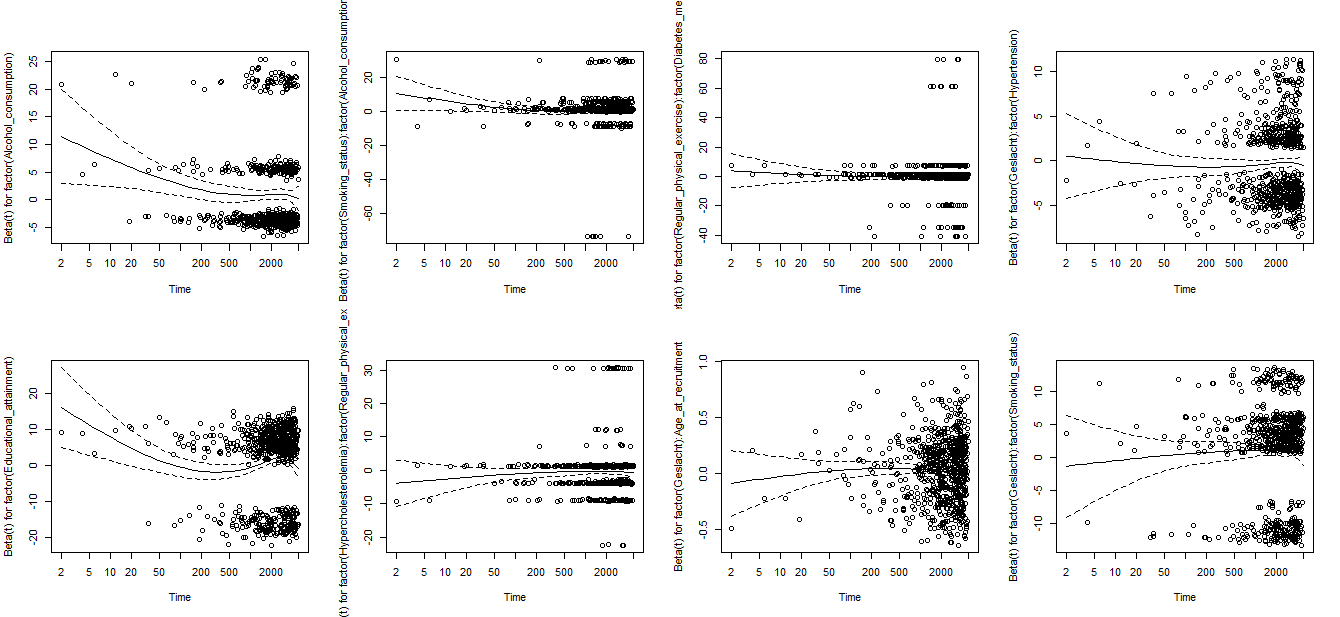
**

| **Supplemental Table 1** **Scaled Schoenfeld residuals tests p-values, tested against different time scales** | | | |
| --- | --- | --- | --- |
| **Predictor** | **Linear** | **Kaplan-Meier-transformed** | **Logarithmic-transformed** |
| Sex | 0.845 | 0.658 | 0.270 |
| Age | 0.010 | 0.010 | 0.027 |
| Family history for stroke | 0.515 | 0.427 | 0.834 |
| Hypertension | 0.595 | 0.549 | 0.479 |
| Smoking status | 0.040 | 0.038 | 0.072 |
| Hypercholesterolemia | 0.890 | 0.933 | 0.826 |
| Regular physical activity | 0.847 | 0.856 | 0.332 |
| DM | 0.411 | 0.477 | 0.303 |
| Alcohol consumption | 0.567 | 0.606 | 0.121 |
| Educational attainment | 0.318 | 0.370 | 0.827 |
| Interactions |  |  |  |
| Smoking status*Alcohol consumption | 0.270 | 0.225 | 0.383 |
| Regular physical activity*Hypercholesterolemia | 0.902 | 0.985 | 0.406 |
| Regular physical activity*DM | 0.949 | 0.919 | 0.428 |
| Sex*Age | 0.751 | 0.942 | 0.142 |
| Sex*Hypertension | 0.872 | 0.714 | 0.651 |
| Sex*Smoking status | 0.122 | 0.094 | 0.450 |
| Global | 0.482 | 0.430 | 0.522 |

DM = diabetes mellitus.

| **Supplemental Table 2** The original regression equation of the SMA^2^SH^2^ERS risk prediction model |
| --- |
| **Linear predictor (LP)**  -0.916 (if woman) +  0.025*age if woman +  0.013*age if man +  0.130 (if family history of stroke) +  -0.292 (if woman and hypertension) +  0.372 (if man and hypertension) +  0.341 (if never smoker and no alcohol consumption) +  0.123 (if never smoker and daily or almost daily alcohol consumption) +  0.281 (if former smoker and no alcohol consumption) +  -0.156 (if woman, former smoker and alcohol consumption on special occasions) +  0.121 (if man, former smoker and alcohol consumption on special occasions) +  -0.085 (if former smoker and daily or almost daily alcohol consumption) +  -0.744 (if current smoker and no alcohol consumption) +  0.430 (if woman, current smoker and alcohol consumption on special occasions) +  0.530 (if man, current smoker and alcohol consumption on special occasions) +  0.021 (if current smoker and daily or almost daily alcohol consumption) +  -0.388 (if regular physical activity and hypercholesterolemia) +  0.658 (if regular physical activity and diabetes mellitus) +  0.017 (if regular physical activity and no hypercholesterolemia or diabetes mellitus) +  -0.022 (if hypercholesterolemia and no regular physical activity) +  -0.352 (if diabetes mellitus and no regular physical exercise) +  0.047 (if low educational attainment) +  -0.173 (if high educational attainment) + |
| **Mean LP**  1.194258 |
