# Supplementary material for: Development and external validation of the SMA2SH2ERS risk prediction model for aneurysmal subarachnoid haemorrhage in the general population: a population-based prospective cohort study
Source: BMJ Open. 2025 Jan 15;15(1):e091756. doi: 10.1136/bmjopen-2024-091756 (PMC11751821; doi:10.1136/bmjopen-2024-091756)
Supplement: Supplementary file 2 [file bmjopen-2024-091756.reviewer_comments.pdf]

PEER REVIEW HISTORY

BMJ Open publishes all reviews undertaken for accepted manuscripts. Reviewers are asked to complete a checklist review form and are provided with free text boxes to elaborate on their assessment. These free text comments are reproduced below.

ARTICLE DETAILS

Title (Provisional)

Development and external validation of the SMA2SH2ERS risk prediction model for aneurysmal subarachnoid hemorrhage in the general population – a population-based prospective cohort study

Authors

Klieverik, Vita; Kanning, Jos P.; Martinsen, Amy E.; Geerlings, Mirjam I.; Ruigrok, Ynte M; Rissanen, Ina; Winsvold, Bendik Slagsvold; Rannikmae, Kristiina

---

VERSION 1 - REVIEW

**Reviewer**	**1**
**Name**	**Van Kammen, Mayte Sánchez**
**Affiliation**	**Amsterdam University Medical Centres**
**Date**	**23-Aug-2024**
**COI**	**I have worked under supervision of the corresponding author 5-10 years ago as a medical student. I now work in a different field and do not believe this constitutes a true competing interest.**

The authors are to be commended on this large study of an interesting research question. Identifying individuals who might benefit from UIA screening could be beneficial for clinical practice and the analysis seems well-performed. However, I think their conclusion that the model performs well in the validation cohort is overstated. The c-statistic was low in both the development and the validation cohort (which might be due to the homogenous population), calibration looks adequate in the development but poor in the validation cohort (although the plots are hard to interpret since the plot extends beyond the depicted range of the y-axis). Although it is hard to develop a well-performing model for such a rare outcome, a low-performing model has no clinical use. I can understand if because the development cohort is much larger, the authors would not want to update the model to better fit the validation cohort. However, they also state the development cohort may actually be less representative of the target population of the model (i.e. primary care patients), due to the overrepresentation of women, older individuals, and persons with higher socioeconomic status in the development cohort. Therefore, I think the overall conclusion of the study

should be modified to something like ‘model fit was inadequate in the validation cohort, future validation in larger cohorts and if necessary adjustment of the model is necessary.’

Please also explain why patients with known UIA were not excluded. It seems including them would increase overall ASAH risk estimations, while in practice the model would not be applied in this patient group.

Some minor comments:

- Please also note the median follow-up time and incidence of ASAH per 100,00 person-years in the validation cohort

- It is stated that the incidence of ASAH in the UK Biobank Cohort was 13.6 per 100,000 person-years and that the incidence of ASAH in the general population is 6.1 per 100,00 person-years. Please elaborate how this twice as high incidence could have affected the results. If applicable, please do the same for the incidence of ASAH in the validation cohort

- Please include a flowchart for each cohort indicating the number of patients excluded due to prior ASAH and the number excluded due to missing data, or mention these numbers in the text

- Please include the calibration plots in the main article, with 95% CIs

- Please include the explanation of variable definitions (e.g. education level, positive family history of stroke, regular exercise) in the main article

Lastly, please also explain more extensively the foreseen practical use of this model. It is stated that individuals with a first-degree relative with ASAH have a 0.4% lifetime risk of ASAH (and a 4% risk of UIA as opposed to the 3% population risk) and UIA screening in these individuals might be cost-effective. It is also stated that the model can identify persons with a 0.52% 10-year risk of ASAH. It is unclear if the same proportion of patients in this group would have UIAs upon screening as those with a first-degree relative with ASAH, and what the rupture risk of these UIAs would be. Based on previous research by the authors it seems that sporadic UIAs have a lower rupture risk than familial UIAs, so it might be that screening this group would just identify a lot of low-rupture-risk UIAs which do not justify preventive treatment but do give stress to the patient. It is also unclear how precise the 0.52% risk estimate is, please add a 95%CI to quantify the uncertainty of this estimate. Lastly, my fear is predicted risks would decrease if patients with known UIA are excluded, non-aneurysmal SAH could be reliably identified and excluded, and generally a cohort with an ASAH incidence more closely resembling the general population is used. Taking together these limitations it seems less likely that the current model could be applied in clinical practice to identify individuals who would benefit from UIA screening. Please discuss these limitations more extensively.

**Reviewer**	**2**
**Name**	**Li , Gaigai**

Affiliation **Tongji Medical College of Huazhong University of Science and Technology**

Date **24-Aug-2024**

COI **-**

The authors aimed to develop and validate a risk prediction model for ASAH in the general population to identify high-risk individuals in this research. They found that SMA2SH2ERS risk prediction model offered insights into ASAH predictors in the general population, identifying individuals with up to a 12-fold increased risk compared to those at lowest risk. The research is novel and clinically relevant, and the methods are appropriate and properly conducted. In addition, the conclusion of the study is clearly expressed and the results are discussed in depth and objectively. As the authors stated, family history of ASAH and ethnicity of the patients should be considered in the development of the risk prediction model. It's better to incorporate some related literature to discuss their possible effects on the incidence of ASAH.

Reviewer **3**

Name **Kelson, Zoe**

Affiliation **University of Exeter, Mathematics**

Date **28-Oct-2024**

COI **None**

This study aimed to develop and validate a risk prediction model for ASAH in the general population to identify high-risk individuals.

Reviewer comments:

"UK Biobank data were used for model development and Trøndelag Health (HUNT) Study data for model validation" [Abstract]

and

"UK Biobank participants are more likely to be women, older individuals, and of higher socioeconomic status compared to non-participants. • Uncertainty on the accuracy of incident ASAH identification in population cohorts like the UK Biobank." [Strengths and limitations of this study]

The authors do well to discuss the representativeness of the cohorts used in this study.

"This study was performed in accordance with the Strengthening the Reporting of Observational Studies in Epidemiology (STROBE) guidelines and the Transparent Reporting of a Multivariable Prediction Model for Individual Prognosis or Diagnosis (TRIPOD) statement. "

Thanks for providing a copy of the associated checklists.

"Candidate predictors, chosen based on literature review, were limited to factors accessible to general practitioners, including sex, age, family history of stroke, hypertension, smoking status, hypercholesterolemia, regular physical activity, hormone replacement therapy (HRT), diabetes mellitus (DM), alcohol consumption, and educational attainment."

A number of valuable and accessible predictors have been included in the study.

It is noted that the authors discuss that "Family history of ASAH was omitted due to unavailability in the UK Biobank."

Can the authors please further discuss other relevant factors that are not readily available for inclusion in the model?

"Statistical analysis involved expressing normally distributed continuous variables as means ± standard deviations (SD) and skewed distributed continuous variables as medians with corresponding interquartile ranges (IQR)"

Can the authors please clarify how they verified distributional assumptions (i.e. was normality checked using visual inspection or statistical tests)?

"Participants with prior ASAH before baseline or missing data were excluded."

and

"In the development cohort, missing data were minimal (ranging from 0.001% to 5.5%) and participants with missing data were excluded. No missing data were observed in the validation cohort."

Can the authors please comment on whether missing data can be considered to be missing at random?

When missing data >5%, say, was a sensitivity analysis to this approach considered (e.g. performing multiple imputation)?

"Interaction terms (age with HRT, smoking with alcohol consumption, regular physical activity with hypertension, hypercholesterolemia, and DM) were included, alongside sex interaction terms with other predictors, to explore potential effect modification by sex"

and

"Using multivariable Cox proportional hazards regression analysis, predictors for ASAH were identified, while correcting for overfitting using bootstrapping techniques." [Abstract]

and

"For model development, multivariable Cox proportional hazards regression analysis was conducted using follow-up time as the time scale... The functional form of continuous candidate predictor age was assessed using martingale residuals. Candidate predictors were considered for model entry regardless of their univariable association with incident ASAH.

Backward selection based on the Akaike Information Criterion (AIC) was performed to evaluate predictor contributions. Proportional hazard assumption was assessed visually and numerically using scaled Schoenfeld residuals plots and tests. To address overfitting, a shrinkage factor was applied to regression coefficients determined by bootstrapping procedures . Hazard ratios (HR) with corresponding 95% confidence intervals (CI) represented the estimated effect sizes of independent predictors ."

Appropriate modelling methods have been applied by the authors.

"Predictive performance was assessed using discrimination and calibration. " [Abstract]

and

"Model performance was evaluated using discrimination and calibration. Discrimination, measured by the concordance statistic (c-statistic), was corrected for overoptimism through bootstrapping. Calibration, an indicator for the agreement between predicted and observed probability of incident ASAH, visually using 5-year and 10-year calibration plots"

The authors satisfactorily assess model performance.

Can other model performance metrics please also be reported, such as sensitvity, specificity, NPV, PPV, accuracy?

Did the authors consider comparing the model performace to other possible models?

Then undertaking a decision curve analysis to help demonstrate the net benefit of the model?

Can the authors please consider exploring how the model performs in subgroups of the population?

"with an online interactive risk calculator developed using the Shiny R package (1.7.1). The risk calculator predicts an individual's absolute ASAH risk at 5- and 10- year follow-up based on provided predictors, with each predictor's contribution calculated by dividing regression coefficients by the smallest coefficient and rounding to the nearest integer"

A valuable resource has been developed by the authors.

Did the authors consider creating high and low risk groups, dividing the population into tertiles or quintiles for example and comparing the prediction of highest and lowest risk groups using Kaplan Meier and Log-rank tests?

"Limitations include missing data in the development cohort, necessitating exclusion of affected participants. However, the small proportion of missing data was anticipated to have minimal impact. Another limitation stems from the use of the UK Biobank as our development cohort, which may skew toward more women, older individuals, and higher socioeconomic status compared to nonparticipants. Nonetheless, we mitigated this by incorporating educational attainment as a proxy for socioeconomic status. Another limitation of the UK Biobank is that it lacked data for familial ASAH so we had to use familial

stroke as a proxy. Moreover, the UK Biobank's limited ethnic diversity precluded subgroup analyses to assess model validity across ethnicities, although validation in the HUNT study, which includes more participants with low education, partially addressed this limitation. Additionally, the accuracy of incident ASAH identification in population cohorts like the UK Biobank remains uncertain, with limited data available. While one study assessing 24 ASAH cases reported a positive predictive value for ASAH ICD codes in the UK Biobank being 71% (95% CI, 49%–87%), further research with larger ASAH patient cohorts is warranted to confirm these findings. Furthermore, uncertainty persists regarding whether the ICD codes used encompass solely ASAH or also include non-aneurysmal cases. We opted to include the ICD-10 I608 and I609 codes, indicative of non-aneurysmal cases, expecting them to account for 10-15% of all subarachnoid hemorrhage cases. However, in our study, their prevalence constituted a significantly larger proportion (418/738, 56.6%), suggesting they likely encompass ASAH cases as well. This supports our decision to include these codes. Sensitivity analysis excluding these ICD-10 I608 and I609 codes yielded a slightly higher c-statistic (0.72 [95% CI, 0.69–0.75] versus 0.62 [95% CI, 0.60–0.64]), albeit with reduced statistical power due to fewer cases. Lastly, the relatively low ASAH incidence contrasts with the prevalent predictors for this disease, which may limit the attainment of a very high cstatistic"

The authors provide a thorough discussion on the main study limitations.

VERSION 1 - AUTHOR RESPONSE

Comments Reviewer 1

1. **The authors are to be commended on this large study of an interesting research question. Identifying individuals who might benefit from UIA screening could be beneficial for clinical practice and the analysis seems well-performed. However, I think their conclusion that the model performs well in the validation cohort is overstated. The c-statistic was low in both the development and the validation cohort (which might be due to the homogenous population), calibration looks adequate in the development but poor in the validation cohort (although the plots are hard to interpret since the plot extends beyond the depicted range of the y-axis). Although it is hard to develop a well-performing model for such a rare outcome, a low-performing model has no clinical use. I can understand if because the development cohort is much larger, the authors would not want to update the model to better fit the validation cohort. However, they also state the development cohort may actually be less representative of the target population of the model (i.e., primary care patients), due to the overrepresentation of women, older individuals, and persons with higher socioeconomic status in the development cohort. Therefore, I think the overall conclusion of the study should be modified to something like 'model fit was inadequate in the validation cohort, future validation in larger cohorts and if necessary adjustment of the model is necessary'.**

➔ First of all, we are happy to hear the positive feedback on our study design and analysis. After going through the model performance results again, we agree that

our final conclusion is overstated. Indeed, it is difficult to develop a well-performing prediction model for a rare outcome, especially when this rare outcome may occur after a very long period of follow-up (since one has to predict the rare outcome even 'farther away' in the future). The calibration of the model in the development cohort is fairly good, but we agree that the calibration of the model in the validation cohort is limited. We already discussed this result to a certain extent in the *Discussion* section on page 12 of our manuscript, but we now decided to move this section to a separate *Future perspectives* section that we added to the *Discussion* section on page 14 and 15 of our manuscript in which we now discuss this result and the implications of our study in more detail:

➔ *"Our SMA^{2}SH^{2}ERS risk prediction model offers insights into ASAH predictors in the general population, identifying individuals with up to a 12-fold increased risk compared to those at lowest risk.*
While the calibration plot demonstrated good correspondence between predicted and observed ASAH risk in the development cohort, there was less good calibration in the validation cohort. It seems that the predictor set in our current risk prediction model is inadequate for accurately predicting ASAH risk in the validation cohort. This discrepancy may arise from differences in ASAH epidemiology and candidate predictors in the validation cohort. Therefore, the SMA^{2}SH^{2}ERS risk prediction model in its current form is not suitable for use in clinical practice. Further validation in larger population-based cohorts and, if necessary, adjustment of the predictor set in the risk prediction model is required. Moreover, prior research indicates the cost-effectiveness of screening for UIAs in individuals with two or more first-degree relatives with ASAH, who have an estimated ASAH lifetime risk of up to 10%. Screening for UIAs is also likely to be cost-effective for those with one first-degree relative with ASAH, who have an estimated ASAH lifetime risk of 0.4%. Given our model's ability to predict 10-year cumulative risks up to 0.52%, it may aid in identifying high-risk individuals for preventive UIA screening. However, future studies must evaluate the cost-effectiveness of such screening for individuals identified as high risk by the SMA^{2}SH^{2}ERS model. Additionally, considering interactions between sex and other predictors, future investigations should explore separate risk prediction models for men and women. Lastly, further research on the differential effects of risk factors and female-specific ASAH risk factors is warranted."

2. **Please also explain why patients with known UIA's were not excluded. It seems including them would increase overall ASAH risk estimation, while in practice the model would not be applied in this patient group.**

➔ We understand the question and we appreciate that the reviewer came up with this issue. We included persons with known UIA's in our analyses because of different reasons. First of all, this ensures that the risk prediction model is representative of the entire general population, as the general population is likely to include persons with both detected and undetected UIA's. Excluding persons with known UIA's would create a biased study sample, potentially reducing the generalizability of the risk prediction model. Secondly, including persons with known UIA's allows the risk prediction model to study risk factors that might interact with UIA presence, such as hypertension or smoking. This may help to refine risk factors that are specifically

associated with UIA rupture, as opposed to refine risk factors that are associated with UIA formation alone.

3. **Please also note the median follow-up time and incidence of ASAH per 100,000 person-years in the validation cohort.**

➔ We know that there were 220 ASAH cases among 46,483 participants in the HUNT Study and we know that the ASAH incidence from a previously published study that used the same HUNT Study was 99 patients during 896,877 person-years of follow-up and therefore 11.0 per 100,000 person-years (*Unruptured intracranial aneurysms in the Norwegian Nord-Trøndelag Health Study (HUNT): risk of rupture calculated from data in a population-based cohort study* by Müller et al [2013]). Therefore, the number of person-years in our validation cohort is estimated at 1,993,060. Consecutively, the incidence of ASAH per 100,000 person-years in our validation cohort is estimated at 11.0 per 100,000 person-years. We added this information to our *Results* on page 10 of our manuscript:
"In the HUNT Study, 46,483 participants were included, with 53.1% women, mean age 59.1 ± 14.1 years, and 220 (0.5%) developed ASAH during an estimated follow-up time of 1,993,060 person-years (11.0 per 100,000 person-years) based on data from a previous study that used the same HUNT Study."

4. **It is stated that the incidence of ASAH in the UK Biobank Cohort was 13.6 per 100,000 person-years and that the incidence of ASAH in the general population is 6.1 per 100,000 person-years. Please elaborate how this twice as high incidence could have affected the results. If applicable please do the same for the incidence of ASAH in the validation cohort.**

➔ We would like to thank the reviewer for this remark as it is certainly a factor that could have affected the results of our risk prediction model. The higher incidence of ASAH in the development cohort may have led to an overestimation of the predicted risks in the risk prediction model. Consecutively, this may lead to a less accurate performance of the risk prediction model if one were to apply it to a population with a lower incidence of ASAH, potentially causing false positives. The estimated incidence of ASAH in the validation cohort is similar to that in the development cohort.
We added these considerations to the *Discussion* section on page 13 of our manuscript:
"The final limitation of using the UK Biobank was that the incidence of ASAH was higher in the development cohort than reported in the general population (13.6 per 100,000 person-years compared to 6.1 per 100,000 person-years, respectively) which may have resulted in an slight overestimation of the predicted risks. Previous research has reported an ASAH incidence of 11.0 per 100,000 person-years in the validation cohort, the HUNT study.[33]"

5. **Please include a flowchart for each cohort indicating the number of patients excluded due to prior ASAH and the number excluded due to missing data, or mention these numbers in the text.**

➔ We already discussed the number of patients excluded from the UK Biobank in the paragraph *Baseline characteristics* of the *Results* section on page 9. Specifically,

36,794 patients were excluded. We chose not to include a flowchart, as it would essentially repeat the details already provided in the text. However, upon reviewing the total number of excluded patients we revisited the reasons for exclusion to determine the separate numbers for patients excluded due to missing data versus prior ASAH. We discovered that the number of patients excluded due to prior ASAH was actually zero. Upon further investigation, we found that prior ASAH was already an exclusion criterium before recruitment of the dataset from the UK Biobank. This means that all data recruited from the UK Biobank were exclusively from patients without prior ASAH. We would like to thank the reviewer for raising this issue, as it prompted us to reevaluate our dataset and correct this oversight. We have updated the first sentence of the paragraph *Baseline characteristics* of the *Results* section on page 9 of our manuscript accordingly:
"From the UK Biobank, 493,650 participants were recruited, with 456,856 included after excluding those with missing data ~~or prior ASAH~~ (n = 36,794)."

1. **Please include the calibration plots in the main article, with 95% CIs.**

➔ We included the calibration plots in the main article. Unfortunately, we were unable to provide the 95% CIs for the calibration plots as the observed and predicted probabilities in both the UK Biobank and the HUNT Study were too small for our statistical software to compute.

2. **Please include the explanation of variable definitions (e.g., education level, positive family history of stroke, regular exercise) in the main article.**

➔ We moved the explanation of the variable definitions from the supplemental materials to the main text under the heading *Outcome and candidate predictors* of the *Methods* section on page 7 of our manuscript:
"A family history of stroke was defined as at least one first-degree relative being affected with the disease. Hypertension was defined as a systolic blood pressure of ≥ 140 mmHg or a diastolic blood pressure of ≥ 90 mmHg and/or use of antihypertensive medication. We categorized smoking status into (1) never smokers, (2) former smokers, and (3) current smokers. Hypercholesterolemia was defined as use of cholesterol lowering medication. Regular physical activity was defined as vigorous physical activity ≥ three times per week. We grouped HRT into (1) never users and (2) former and current users combined. DM was defined based on a past medical history of DM and/or use of antidiabetic medication. We categorized alcohol consumption into (1) no alcohol consumption, (2) alcohol consumption on special occasions, and (3) daily or almost daily alcohol consumption. We grouped educational attainment into (1) high, (2) intermediate, and (3) low educational attainment. High educational attainment was defined as having a university or college degree, intermediate educational attainment as having either an Advanced Level qualification, Ordinary Level qualification, Certificate of Secondary Education (CSE), National Vocational Qualification (NVQ), Higher National Diploma (HND), Higher National Qualification (HNC), or other professional qualification, and low educational attainment as having no degree."

3. **Lastly, please also explain more extensively the foreseen practical use of this model. It is stated that individuals with a first-degree relative with ASAH have a**

0.4% lifetime risk of ASAH (and a 4% risk of UIA as opposed to the 3% population risk) and UIA screening in these individuals might be cost-effective. It is also stated that the model can identify persons with a 0.52% 10-year risk of ASAH. It is unclear if the same proportion of patients in this group would have UIAs upon screening as those with a first-degree relative with ASAH, and what the rupture risk of these UIAs would be. Based on previous research by the authors it seems that sporadic UIAs have a lower rupture risk than familial UIAs, so it might be that screening this group would just identify a low of low-rupture-risk UIAs which do not justify preventive treatment but do give stress to the patient. It is also unclear how precise the 0.52% risk estimate is, please ad a 95% CI to quantify the uncertainty of this estimate. Lastly, my fear is predicted risks would decrease if patients with known UIA are excluded, non-aneurysmal SAH could be reliably identified and excluded, and generally a cohort with an ASAH incidence more closely resembling the general population is used. Taking together these limitations it seems less likely that the current model could be applied in clinical practice to identify individuals who would benefit from UIA screening. Please discuss these limitations more extensively.

We would like to thank the reviewer for this extensive comment and we understand the concerns regarding the clinical use of this risk prediction model. We agree that the SMA^2SH^2ERS risk prediction model in its current form is not suitable for use in clinical practice. We also addressed this point in the answer to the first comment of the reviewer and we would like to refer to our response we gave there.

We appreciate that the reviewer extensively considered whether the same proportion of individuals in the general population would have UIAs upon screening as those with a first-degree relative with ASAH, and what the rupture risk of these UIAs would be. We agree with the reasoning that, because the prevalence of UIAs in the general population is lower than in persons with a first-degree relative with ASAH, and because the risk of rupture of sporadic UIAs is lower than that of familial UIAs, one might expect that screening for UIAs in the general population would primarily identify UIAs with a low rupture risk. However, our results show that the SMA^2SH^2ERS score has the ability to predict a 10-year cumulative risk of ASAH up to 0.52%, which exceeds the lifetime cumulative risk of ASAH in individuals with a first-degree relative with ASAH (0.4%). This finding suggests that there may be individuals in the general population with a higher cumulative risk of ASAH than those with a familial predisposition, despite the above reasoning. Whether screening for UIAs in these high-risk individuals in the general population is cost-effective needs to be evaluated in future studies.

Furthermore, we added the 95% CIs to the predicted risk estimates to the section under the heading Individual risk prediction of the Results section on page 11 of our manuscript:

"The mean predicted 5-year absolute ASAH risk was 0.05% (95% CI 0.0498%–0.0503%), ranging from 0.018% (95% CI 0.016%–0.021%) to 0.22% (95% CI 0.15%–0.29%). Predicted 10-year cumulative absolute risk averaged 0.13% (95% CI 0.129%–0.131%), ranging from 0.042% (95% CI 0.041%–0.044%) to 0.52% (95% CI 0.35%–0.68%)."

Comments Reviewer 2

1. **The authors aimed to develop and validate a risk prediction model for ASAH in the general population to identify high-risk individuals in this research. They found that SMA^{2}SH^{2}ERS risk prediction model offered insights into ASAH predictors in the general population, identifying individuals with up to a 12-fold increased risk compared to those at lowest risk. The research is novel and clinically relevant, and the methods are appropriate and properly conducted. In addition, the conclusion of the study is clearly expressed and the results are discussed in depth and objectively. As the authors stated, family history of ASAH and ethnicity of the patients should be considered in the development of the risk prediction model. It's better to incorporate some related literature to discuss their possible effects on the incidence of ASAH.**

➔ We are happy to hear the reviewer's positive feedback on our study. We agree with the reviewer on the point that family history of ASAH and ethnicity should be considered in the development of the risk prediction model. This was discussed as such in the paragraph on *Strengths and limitations* of the *Discussion* section on page 13 and 14 of our manuscript:
"Another limitation of the UK Biobank is that it lacked data for familial ASAH so we had to use familial stroke as a proxy. Moreover, the UK Biobank's limited ethnic diversity precluded subgroup analyses to assess model validity across ethnicities, although validation in the HUNT study, which includes more participants with low education, partially addressed this limitation."

In various sections of our manuscript, we have already referenced to literature showing that both family history of ASAH and ethnicity are risk factors for ASAH (*Bor et al 2008*, *Bor et al 2014*, *Feigin et al 2005*, *Greving et al 2014* and *Etminan et al 2019*). Based on these findings, we believe that incorporating both family history of aSAH and ethnicity into the SMA^{2}SH^{2}ERS model would likely enhance its predictive performance and generalizability across different ethnic populations, We included this observation, along with references to the relevant literature, in the paragraph on *Strengths and limitations* of the *Discussion* section on page 13 and 14:
"Another limitation of the UK Biobank is that it lacked data for familial ASAH so we had to use familial stroke as a proxy. This may have reduced the predictive performance of the model, as family history of ASAH is a known risk factor for ASAH.[10,12] Moreover, the UK Biobank's limited ethnic diversity precluded subgroup analyses to assess model validity across ethnicities, which may have reduced the generalizability of our model.[3,13,14] However, validation in the HUNT study, which includes more participants with low education, partially addressed this limitation.[18]"

Comments Reviewer 3

1. **It is noted that the authors discuss that "Family history of ASAH was omitted due to unavailability in the UK Biobank."**
   Can the authors please further discuss other relevant factors that are not readily available for inclusion in the model?

➔ We agree that there may be other relevant factors to include in the risk prediction model that were unavailable in the development and validation cohorts. We believe that the most important of those unavailable relevant factors would be family history of ASAH and ethnicity. We discussed these two factors in the paragraph *Strengths and limitations* of the *Discussion* section on page 13 and 14 of our manuscript:

"Another limitation of the UK Biobank is that it lacked data for familial ASAH so we had to use familial stroke as a proxy. <u>This may have reduced the predictive performance of the model, as family history of ASAH is a known risk factor for ASAH.[10,12]</u> Moreover, the UK Biobank's limited ethnic diversity precluded subgroup analyses to assess model validity across ethnicities, <u>which may have reduced the generalizability of our model.[3,13,14] However,</u> validation in the HUNT study, which includes more participants with low education, partially addressed this limitation.[18]"

2. **Statistical analysis involved expressing normally distributed continuous variables as means ± standard deviations (SD) and skewed distributed continuous variables as means with corresponding interquartile ranges (IQR)."**
   Can the authors please clarify how they verified distributional assumptions (i.e., was normality checked using visual inspection or statistical tests)?

➔ We would like to thank the reviewer for reminding us that we should clarify how we verified the distributional assumptions. We did so visually, so we checked normality of all variables by visually inspecting normal probability plots. We included this in the paragraph *Statistical analysis* of the *Methods* section on page 8 of our manuscript:
"<u>Distributional assumptions were verified visually using normal probability plots.</u>"

3. **"Participants with prior ASAH before baseline or missing data were excluded."**
   And
   "In the development cohort, missing data were minimal (ranging from 0.001% to 5.5%) and participants with missing data were excluded. No missing data were observed in the validation cohort."
   Can the authors please comment on whether missing data can be considered to be missing at random? When missing data >5%, say, was a sensitivity analysis to this approach considered (e.g., performing multiple imputation)?

➔ We believe that the missing data in our development cohort is either Missing Completely At Random (MCAR) or Missing at Random (MAR). MCAR indicates that the missing data is entirely unrelated to the variables or the outcome. We consider this is the most likely option because the data in the UK Biobank was collected from registries, electronic health records, and individuals invited to a research center for measurements. When some variables are missing because individuals were unable

to visit a research for measurements, this constitutes MCAR. MAR, on the other hand, occurs when certain data is missing in a specific subgroup, such as when hypertension data is missing for individuals who smoke. Give these considerations and of the fact that the percentage of missing data was very small, we decided not to perform multiple imputation to address the missing data.

4. **"Predictive performance was assessed using discrimination and calibration." [Abstract]**
   And
   "Model performance was evaluated using discrimination and calibration. Discrimination, measured by the concordance statistic (c-statistic), was corrected for overoptimism through bootstrapping. Calibration, an indicator for the agreement between predicted and observed probability of incident ASAH, visually using 5-year and 10-year calibration plots."
   The authors satisfactorily assess model performance. Can other model performance metrics please also be reported, such as sensitivity, specificity, NPV, PPV, accuracy?

➔ We really appreciate that the reviewer suggested metrics for model performance with the aim to improve the reporting of our model. However, we intentionally did not calculated a sensitivity, specificity, NPV, PPV and accuracy as these are performance metrics for diagnostics model and our model is a prognostic model. This means that these performance metrics do not apply to our model.

5. **Did the authors consider comparing the model performance to other possible models?**

➔ We did consider comparing our model performance to other similar models. However, unfortunately, there are no other similar models to which we could have compared our model performance.

6. **Then undertaking a decision curve analysis to help demonstrate the net benefit of the model?**

➔ We believe that assessing the net benefit of using the SMA^{2}SH^{2}ERS risk prediction model indeed is essential before it can be used in clinical practice. However, assessing this net benefit was out of the scope of this study. We believe that the net benefit of the SMA^{2}SH^{2}ERS score depends on its effectiveness of preventing ASAH by identifying high-risk individuals with UIAs and deciding whether they need preventive treatment of their UIAs or not, as well as the risks and cost-effectiveness. We already discussed this last point under the heading *Future perspectives* of the *Discussion* section on page 15 of our manuscript:
"However, future studies must evaluate the cost-effectiveness of such screening for individuals identified as high risk by the SMA^{2}SH^{2}ERS model."
We now also added the statement that assessing the net benefit of using the SMA^{2}SH^{2}ERS risk prediction model before it can be used in clinical practice to the *Future perspectives* section of the *Discussion* section on page 15 of our manuscript:
"However, future studies must evaluate the cost-effectiveness <u>as well as the net benefit</u> of such screening for individuals identified as high risk by the SMA^{2}SH^{2}ERS model."

7. **Can the authors please consider exploring how the model performs in subgroups of the population?**

➔ We appreciate that the reviewer came up with this comment as we believe this is a very important research question for future research. In fact, we already started on assessing how well the model performs in men and women separately and we hopefully have a paper coming out on this topic soon.

8. **Did the authors consider creating high and low risk groups, dividing the population into tertiles or quintiles for example and comparing the prediction of highest and lowest risk groups using Kaplan Meier and Log-rank tests?**

➔ Yes, we considered creating high and low risk groups based on the predicted generated by our model. However, we ultimately decided against this grouping for two primary reasons. First the event rate of ASAH in our cohorts presented a challenge. For rare outcomes like ASAH, it is crucial to have a sufficient number of events in each risk group to ensure reliable estimates. If certain risk group contain too few events, this can reduce the statistical power of the log-rank test and result in unreliable risk stratification. Second, there is temporal variation in risk. The risk of ASAH may vary over time, and static risk groups do not account for such temporal dynamics. Ideally, this type of grouping would require the incorporation of time-varying covariates to capture these changes more accurately. We hope this explanation clarifies why we opted not to create high and low risk groups in our study.
